# A comprehensive SARS-CoV-2 and COVID-19 review, Part 2: host extracellular to systemic effects of SARS-CoV-2 infection

**DOI:** 10.1038/s41431-023-01462-1

**Published:** 2023-11-08

**Authors:** S. Anand Narayanan, David A. Jamison, Joseph W. Guarnieri, Victoria Zaksas, Michael Topper, Andrew P. Koutnik, Jiwoon Park, Kevin B. Clark, Francisco J. Enguita, Ana Lúcia Leitão, Saswati Das, Pedro M. Moraes-Vieira, Diego Galeano, Christopher E. Mason, Nídia S. Trovão, Robert E. Schwartz, Jonathan C. Schisler, Jordana G. A. Coelho-dos-Reis, Eve Syrkin Wurtele, Afshin Beheshti

**Affiliations:** 1 COVID-19 International Research Team, Medford, MA 02155 USA; 2 Department of Health, Nutrition and Food Sciences, Florida State University, Tallahassee, FL 32301 USA; 3 Center for Mitochondrial and Epigenomic Medicine, Division of Human Genetics, The Children’s Hospital of Philadelphia, Philadelphia, PA 19104 USA; 4 Center for Translational Data Science, University of Chicago, Chicago, IL 60637 USA; 5 Clever Research Lab, Springfield, IL 62704 USA; 6 Departments of Oncology and Medicine and the Sidney Comprehensive Cancer Center, The Johns Hopkins Medical Institutions, Baltimore, MD USA; 7 Human Healthspan, Resilience, and Performance, Florida Institute for Human and Machine Cognition, Pensacola, FL 32502 USA; 8 Sansum Diabetes Research Institute, Santa Barbara, CA 93015 USA; 9 Department of Physiology, Biophysics and Systems Biology, Weill Cornell Medicine, New York, NY USA; 10 Laboratory of Virology and Infectious Disease, The Rockefeller University, New York, NY 10065 USA; 11 Cures Within Reach, Chicago, IL 60602 USA; 12 Campus and Domain Champions Program, Multi-Tier Assistance, Training, and Computational Help (MATCH) Track, National Science Foundation’s Advanced Cyberinfrastructure Coordination Ecosystem: Services and Support (ACCESS), Philadelphia, PA USA; 13 Expert Network, Penn Center for Innovation, University of Pennsylvania, Philadelphia, PA 19104 USA; 14 Biometrics and Nanotechnology Councils, Institute for Electrical and Electronics Engineers, New York, NY 10016 USA; 15 Peace Innovation Institute, The Hague 2511, Netherlands and Stanford University, Palo Alto, 94305 CA USA; 16 Instituto de Medicina Molecular João Lobo Antunes, Faculdade de Medicina, Universidade de Lisboa, 1649-028 Lisboa, Portugal; 17 MEtRICs, Department of Chemistry, NOVA School of Science and Technology, Universidade NOVA de Lisboa, 2829-516 Caparica, Portugal; 18 Atal Bihari Vajpayee Institute of Medical Sciences and Dr Ram Mannohar Lohia Hospital, New Delhi, 110001 India; 19 Department of Genetics, Microbiology and Immunology, Institute of Biology, University of Campinas, Campinas, Brazil; 20 Experimental Medicine Research Cluster (EMRC) and Obesity and Comorbidities Research Center (OCRC), University of Campinas, Campinas, Brazil; 21 Facultad de Ingeniería, Universidad Nacional de Asunción, San Lorenzo, Paraguay; 22 New York Genome Center, New York, NY USA; 23 The Feil Family Brain and Mind Research Institute, Weill Cornell Medicine, New York, NY USA; 24 Fogarty International Center, National Institutes of Health, Bethesda, MD 20892 USA; 25 Division of Gastroenterology and Hepatology, Department of Medicine, Weill Cornell Medicine, New York, NY USA; 26 Department of Physiology and Biophysics, Weill Cornell Medicine, New York, NY USA; 27 McAllister Heart Institute and Department of Pharmacology, The University of North Carolina at Chapel Hill, Chapel Hill, NC USA; 28 Basic and Applied Virology Lab, Department of Microbiology, Institute for Biological Sciences (ICB), Federal University of Minas Gerais, Belo Horizonte, MG Brazil; 29 Genetics Program, Department of Genetics, Development and Cell Biology, Iowa State University, Ames, IA 90011 USA; 30 Bioinformatics and Computational Biology Program, Center for Metabolomics, Department of Genetics, Development and Cell Biology, Iowa State University, Ames, IA 90011 USA; 31 Stanley Center for Psychiatric Research, Broad Institute of MIT and Harvard, Cambridge, MA 02142 USA; 32 Blue Marble Space Institute of Science, Space Biosciences Division, NASA Ames Research Center, Moffett Field, Santa Clara, CA 94035 USA

**Keywords:** Viral infection, Diagnostic markers, Pathology, Chronic inflammation

## Abstract

COVID-19, the disease caused by SARS-CoV-2, has caused significant morbidity and mortality worldwide. The betacoronavirus continues to evolve with global health implications as we race to learn more to curb its transmission, evolution, and sequelae. The focus of this review, the second of a three-part series, is on the biological effects of the SARS-CoV-2 virus on post-acute disease in the context of tissue and organ adaptations and damage. We highlight the current knowledge and describe how virological, animal, and clinical studies have shed light on the mechanisms driving the varied clinical diagnoses and observations of COVID-19 patients. Moreover, we describe how investigations into SARS-CoV-2 effects have informed the understanding of viral pathogenesis and provide innovative pathways for future research on the mechanisms of viral diseases.

## Introduction

As of January 13, 2023, over 671 million cases of the coronavirus disease 2019 (COVID-19) worldwide have been reported, and more than 6.71 million lives have been claimed globally. The COVID-19 pandemic is caused by infection with the severe acute respiratory syndrome coronavirus 2 (SARS-CoV-2) virus. The development and administration of vaccines and antiviral therapeutics against SARS-CoV-2 significantly reduced the local to global impact and burden of COVID-19. However, the global distribution of these resources has been unequal [1], ultimately prolonging viral transmission. Moreover, SARS-CoV-2 quickly evolved into variants with increased transmission rates more capable of immune escape [2]. Therefore, we must increase our understanding of this novel virus, its variants, and the resulting short- and long-term effects of COVID-19 on human health.

SARS-CoV-2 belongs to the genus Betacoronavirus, which includes viruses such as OC43, HKU1, SARS-CoV, and MERS-CoV [3]. SARS-CoV-2 is an enveloped positive-sense RNA virus, whose genome is packaged in a helical ribonucleocapsid complex. The outer surface of the virus is studded with Spike (S) proteins that facilitate the infection of host cells by binding to its cognate cellular surface receptor angiotensin-converting enzyme 2 (ACE2) through the S protein receptor binding domain (RBD) to allow for viral membrane fusion. Fusion leads to viral genome transfer into the host cell cytoplasm where viral replication can commence, leading to its various intracellular impacts. To read in more detail these processes, as well as structural elements of the SARS-CoV-2 virus, see our first review, ref. [3], focusing on intracellular SARS-CoV-2 viral effects [3].

In this second of a three-part comprehensive series of our series of SARS-CoV-2 reviews, we cover the extracellular and circulating signals and physiological effects of the viral disease, including the consequences and remodeling of organs, metabolism, humoral factors, and the immune system. In turn, the aim of this review series is to provide an overview of our current knowledge of SARS-CoV-2 and its biological effects from basic biology to clinical patient outcomes, highlighting also future areas of research given the ever-evolving nature of SARS-CoV-2,COVID-19, and its consequences for healthcare across the world.

## Metabolic adaptations caused directly by viral infection of cells

We previously described [3] SARS-CoV-2 viral infection intracellular effects and host adaptations. These include major shifts in metabolic and biochemical pathways, including the oxidative phosphorylation and the tricarboxylic acid (TCA) cycle [3]. The immune system is also involved in these adaptations, with a study showing upregulation of Type I interferon (IFN) gene signaling pathways leading to changes with cellular responses to IFN and triggering changes with associated cytokine-mediated signaling pathways [4]. These stimuli lead to cellular adaptations including endoplasmic reticulum stress, upregulated unfolded protein response and downregulation of pathways including spliceosome-mediated RNA processing, mitochondrial electron transport chain inhibition, and impairment in the biogenesis and assembly of the electron transport chain complexes and oxidative phosphorylation [5, 6]. Indeed, SARS-CoV-2 infection leads to decreased mitochondrial function, increased glycolysis, and shunting towards the pentose 5 phosphate pathway, resulting in changes in cellular metabolism and bioenergetics that lead to pathophysiological outcomes.

These various cellular biochemical adaptations also result in production of circulating metabolites in COVID-19 patients that lead to systemic effects, including tissue remodeling and function, shifts in whole-body metabolism and bioenergetics, changes with immune and endocrine state, etc., resulting in the symptoms and disease outcomes seen with COVID-19 patients. For example, the TCA cycle is the main cellular source of energy and critical for aerobic respiration; serum levels of the TCA metabolites (citrate, fumarate, malate, and aconitate) were significantly lower in the SARS-CoV-2-infected patients as compared with controls, with choline specifically observed to be decreased in patients with severe COVID-19 [7]. Serum metabolites that were increased in these patients included deoxy-thymidine, deoxyuridine, adenine, cystine, and homocysteine. These data provide context as to how specific biochemical and physiological pathways, e.g., the TCA cycle, shift as a result of COVID-19 infection and lead to pathogenesis. For example, elevated homocysteine is associated with endothelial damage [8]. These shifts in metabolites reflect a systemic shift during acute COVID-19 infection, and warrant study of subsequent specific organ adaptations (e.g., bone as a major source of citrate, which is released into the plasma during bone resorption, with soft tissues not generally providing a significant source of citrate into blood plasma).

Separate from metabolic pathways (e.g., oxidative phosphorylation, glycolysis, etc.) that produce energy from sugars are alternative pathways that utilize compounds such as lipids. Excess energy is stored in the form of lipids, with lipids also used as an immediate energy source or for later use. Lipids also serve as second messengers, activating protein and immune pathways. Indeed, it has been reported SARS-CoV-2 infection causes lipids levels to change and lipid metabolism to alter, with one study using untargeted metabolomic and lipidomic approaches observing specific circulating lipids associated with SARS-CoV-2 infection, including increases in triglycerides and free fatty acids (e.g., arachidonic acid and oleic acid). These lipids also correlated with disease severity [9]. A specific class of lipids are lipid mediators (LMs), which are bioactive lipids produced locally in response to extracellular stimuli and are involved in immune regulation. Studies have investigated their role in relation to COVID-19 severity as well. One report involving 19 healthy patients, 18 COVID-19 patients who did not need ICU admission (mild), and 20 patients that needed ICU admission (severe) showed an increase of free poly-unsaturated fatty acids (PUFAs) and diminished amounts of PUFA-containing plasmalogens in patients with COVID-19 that also correlated with disease severity [10]. Another study performing metabolomics and lipidomics measurements on patients (49 subjects, 33 were COVID-19–positive subjects and 16 COVID-19–negative subjects) showed alterations in fatty acid metabolism that were associated with SARS-CoV-2 infection. More specifically, short- and medium-chain acylcarnitines were significantly diminished in all patients with COVID-19, while all Non-Esterified Fatty Acids (except nonanoic acid) were increased in all COVID-19 patients [11, 12].

In summary, SARS-CoV-2 infection affects whole-body metabolism in different ways, with emerging evidence supporting a correlation between these adaptations and COVID-19 disease severity. Moreover, the balance of biochemical components, whether the machinery (e.g., TCA proteins), fuel sources (e.g., lipids), or messenger molecules (e.g., metabolites) appear to shift towards a physiological catabolic state that may be further exacerbated by a patient’s underlying conditions and demographics [7, 9–12]. Metabolic biofactors are also intrinsically tied with the recovery and response to viral infection, with immune-regulating lipids, pro-inflammatory lipids, and lipid mediators plausibly modulating the immune response during COVID-19, as one example. The immune system’s physiology is also reliant on its metabolic function, whether from a development, proliferation, activation, or memory perspective. Immunity is shaped and guided by humoral factors, which we address in the next section, as to the SARS-CoV-2 viral influence on these factors and the effects on the body.

## Humoral adaptations and changes

Humoral factors are compounds that are transported by the circulatory system throughout the body, and include immune factors (e.g., cytokines, chemokines, antibodies, complement proteins, clotting factors, etc.), hormones, RNAs, lipids, metabolites, etc. These play a role in steady-state physiology as well as pathophysiological adaptations, with changes in humoral factors occurring from SARS-CoV-2 infection. One example is bradykinin, a peptide that is involved with physiological effects, including dilation of arterioles through release of prostacyclin, nitric oxide, and EDHF, constriction of veins by prostaglandin F2, as well as various biochemical effects including upregulation of intracellular Ca2+, regulation of cAMP/cGMP, arachidonic acid release, promotion of VEGF expression and angiogenesis, and activation of TGF-B, JAK/STAT pathways [13]. Additionally, bradykinin is degraded by angiotensin-converting enzyme (ACE) and enhanced by angiotensin produced by ACE2. Bradykinin, angiotensin, ACE, and ACE2, are all part of the renin-angiotensin system (RAS), which serves a role within the endocrine system in regulation of renal, cardiac, and vascular physiology, and in turn, systemic physiology [14]. Moreover, SARS-CoV-2 enters the cell through receptors such as ACE2 and NRP-1, in a process requiring the transmembrane protease 2 (TMPRSS2) protein [3, 15]; thus, SARS-CoV-2 directly interacts with the RAS system and influences its activity. Indeed, gene analysis of bronchoalveolar lavage fluid from COVID-19 patients showed a critical imbalance of RAS-related factors, including decreased expression of ACE, while increases in ACE2, renin, angiotensin, and bradykinin, which were suggestive of elevated bradykinin levels systemically that lead to the cardiovascular symptoms seen with patients [16]. Another study of the bradykinin cascade of COVID-19 patients correlated changes in this pathway with disease severity, showing accumulated bradykinin levels were related with patient disease severity, as well as with levels of inflammation, coagulation, and lymphopenia [17].

In addition to the RAS axis, the endocrine system is also vulnerable to SARS-CoV-2 infection [14, 15]. For example, the ACE2 receptor is expressed in endocrine glands, including the hypothalamus, pituitary gland, adrenals, pancreas, thyroid glands, ovaries, and testes [15]. Studies have shown reduced thyroid (e.g., subacute thyroiditis, thyrotoxicosis), adrenal (e.g., hypocortisolism, hyponatremia), hypothalamic-pituitary-thyroid (e.g., HPA dysfunction, hypocortisolism, hypothyroidism), and pancreatic islet function (e.g., hyperglycemia, pancreatic injury based from elevated amylase and lipase), as well as damage to the testes and changes with menstrual cycle being described in COVID-19 patients [14, 15]. SARS-CoV-2 infection has also been suggested to affect glycemic control, with an international registry having been organized to investigate the interaction between diabetes and COVID-19 [14]. Moreover, clinical studies show more severe outcomes in patients with diabetes, obesity, and hypertension; however, there is a lack of data in humans on ACE2 expression in pathological conditions with endocrine tissues, an area which needs future investigation [15]. Various mouse studies have enabled us to investigate organ-specific adaptations, including examining the effects of ACE2 deletion in endocrine organs and observing the beneficial effects of ACE2 gene therapy [15]. The details of these studies are outside the scope of this review, but are elaborated in ref. [14, 15]. As with bradykinin, the remodeling of endocrine factors results in changes with the factors that endocrine organs produce and secrete, though this is an area warrants more investigation. Indeed, these include the non-coding transcriptome, genes which are not involved with protein synthesis, but are transcribed and often secreted into the circulation, in turn providing information on the genetic state of their originating cells, but also controlling and regulating biological adaptations due to the ability of the non-coding RNAs to interact with other macromolecules, including DNA, RNA, and proteins. We elaborate on the changes with the non-coding RNA transcriptome seen in COVID-19 patients in the next section.

## Exosomes influence on SARS-CoV-2 infection

Exosomes are extracellular vesicles generated by all cells that carry cellular components, including nucleic acids, proteins, lipids, and metabolites. They are carried and transported in blood and lymph, and serve near- and long-distance intercellular communication and biological effects. They also serve as a biomarker based on their contents, which may be altered in different conditions and physiological states. It is known that during viral infection, extracellular vesicles become vectors of viral material. More recent reports have also highlighted this occurs with SARS-CoV-2 infection [18].

One study investigated and compared exosomes from mild or severe COVID-19 patients. The exosomes from both groups contained SARS-CoV-2 spike-derived peptides and immunomodulatory molecules, though only those from mild patients could stimulate an antigen-specific CD4 T-cell response [19]. This study also observed that the proteome of exosomes of mild patients correlated with a normally functioning immune system, while that of severe patients was associated with increased and chronic inflammation.

As mentioned above, exosomes may affect distant cells, and one study explored this connection in the context of COVID-19 by evaluating exosome effects on endothelial cells from exosomes derived from plasma [19]. Exosomes affected the endothelial cells by inducing inflammation-related pathways (e.g., NRLP3, caspase-1, and interleukin-1-beta) through mRNA expression, compared with plasma from mild disease or healthy donors. The study showed that these adaptations were tied with exosomes containing tenascin-C and fibrinogen-beta, which triggered the inflammatory pathways by inducing NF-kB mediated pathways. While this study focused on endothelial cell responses, it was noted that these effects may promote inflammation in other cell types systemically.

Exosomes can also be involved with the inflammatory response. A subset of exosomes, called defensosomes, mediate host defense by binding and inhibiting pathogenic responses. A study showed that exosomes in SARS-CoV-2-infected patients can also be involved in this capacity by interfering with surface protein interactions [20]. Exosomes containing high levels of the viral receptor, ACE2, were induced from SARS-CoV-2 infection and activation of viral sensors in cell culture utilizing the autophagy protein, ATG16L1, and were shown to be able to bind and block viral entry. These findings support that exosomes may also be involved with the SARS-CoV-2 antiviral response, with more research of this area increasing our understanding of exosomes and their role in host defense mechanisms.

Finally, exosomes may provide insight into organ adaptations based on exosomes from the source organ, in general as well as in specific circumstances such as from viral infection. The exosomes produced from specific cells will communicate their cellular phenotypic state, with each exosome sharing the identity of its source cell. In turn, organ-specific adaptations from SARS-CoV-2 infection may be able to be delineated through measurement of exosomes. One study explored this by investigating how SARS-CoV-2 is known to invade neural cell mitochondria, with SARS-CoV-2 proteins and mitochondrial proteins contained within neuron- and astrocyte-derived exosomes quantified in post COVID-19 infection. Indeed, these exosomes contained SARS-CoV-2 N and S1 proteins and varied depending on the context of the disease severity and timeline of COVID-19 [21]. This study is one example of the utility exosomes may provide for complex, multi-organ conditions like COVID-19, in studying and determining physiological alterations resulting from the disease condition.

## Roles of the non-coding transcriptome in SARS-CoV-2 infection

**Fig. 1:**
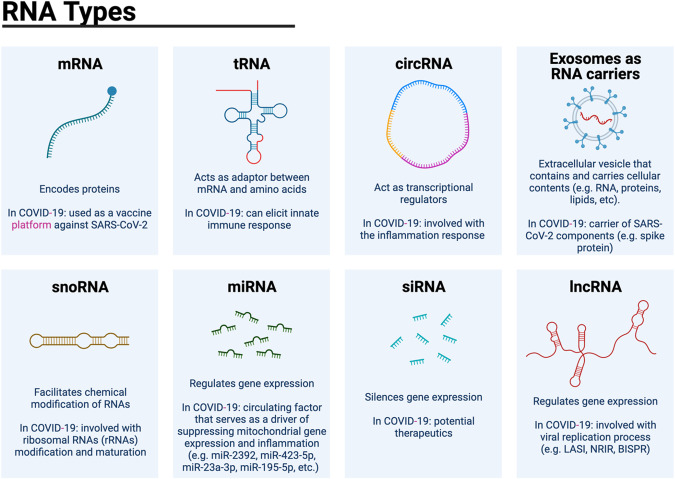
Different RNA and their role in COVID-19. Structure and description of different categories of RNA involved in COVID-19.

RNA viruses such as SARS-CoV-2 are flexible in their ability to circumvent cellular defenses through a variety of mechanisms that often involve specific interactions with cellular components. Additionally, some viral genomes can also be processed by cellular machinery to generate non-coding RNAs (ncRNAs) that could have regulatory functions. Among them, specific elements of the host ncRNA transcriptome include microRNAs (miRNAs), long non-coding RNAs (lncRNAs) and circular RNAs (circRNAs), have been described as important players in the regulation of the viral replicative cycle and the cellular antiviral responses (Fig. 1). To summarize the current knowledge about ncRNAs and SARS-CoV-2, we share the latest details of the different ncRNA families and their regulatory roles during infection and disease progression.

## miRNAs

We begin with highlighting microRNAs (miRNAs), which are short regulatory non-coding RNAs generated by the enzymatic processing of specific RNA transcripts. Their regulatory functions at the cellular level are exerted by recruiting regulatory proteins (RNA-induced silencing complex, or RISC) which are further targeted to the 3′-UTR of mRNAs by base complementarity, causing a repressive effect on gene expression [22]. Considering the possible involvement of dysregulated miRNAs upon infection on the systemic manifestations of COVID-19, multiple research groups profiled circulating miRNA levels in SARS-CoV-2 patients. One study of a stratified cohort of COVID-19 patients showed a molecular signature composed of three blood-circulating miRNAs (hsa-miR-423-5p, hsa-miR-23a-3p and hsa-miR-195-5p), independently classified COVID-19 cases, distinguishing them from other viral infections such as H1N1 influenza and healthy controls [23].

Recently a circulating miRNA, hsa-miR-2392, was shown and suggested to be one of the main drivers of a suppressive effect of mitochondrial gene expression and inflammation effectors, promoting many of the described symptoms associated with COVID-19. This miRNA was also detected in SARS-CoV-2-infected patient serum and urine, opening the possibility of its use as a prognostic biomarker for the disease [24]. Mitochondrial analysis showed miR-2392 targets were OXPHOS, mitochondrial translation, and mitochondrial metabolism related transcripts. In summary, SARS-CoV-2 elevates miRNAs, such as miR-2392, which can result in affecting biochemical pathways (e.g., downregulation of OXPHOS), and treatment with anti-miR-2393 therapeutics potentially blocks SARS-CoV-2 infection in hamsters and hACE2-A549 cells [24].

Several additional miRNA’s have also been observed to be involved with COVID-19 outcomes (e.g., level of viral load, biomarkers, etc.), as well as having various biochemical roles (e.g., viral entry, cytokine storm development, activation of signaling pathways, etc.). These include miRNA interaction with cell death processes, regulating pathways such as p53 by miR-101, miR-100, miR-7, miR-107, etc., as well with the viral entry process (e.g., hsa-miR-98-5p binding to TMPRSS2 transcript). Additionally, cellular inflammation processes in COVID-19 infected individuals are affected by miRNA’s (e.g., miR-146a, miR-21, and miR-142), by promoting MAPK and NF-kB signaling causing cellular pro-inflammatory phenotypic adaptations, which has additional consequences such as mitochondrial stress from production of inflammatory factors and reactive oxygen species. These highlight the significant and diverse roles miRNA’s play in steady-state physiology and viral infection induced pathophysiology in COVID-19, which here we briefly describe, but are covered in more detail in the following review [25].

## lncRNAs

Long non-coding RNAs (lncRNAs) are a wide family of non-coding transcripts characterized by their lack of coding potential, their larger sizes when compared to other ncRNAs (more than 200 nucleotides), and their origin from devoted transcriptional units. Functionally, lncRNAs can regulate the dynamics of the genomic output from chromatin to the cytoplasm, mainly by acting as molecular scaffolds of biological complexes that establish interactions with proteins, DNA, and other RNA molecules. Early transcriptomic studies using cell models show SARS-CoV-2 infection induces a transcriptional pattern that produces several differentially expressed lncRNAs [26]. Through co-expression network analysis, another study identified four differentially expressed lncRNAs correlating with genes involved in various immune-related pathways, suggesting that the aberrant expression of these four lncRNAs can be associated with SARS-CoV-2 cytokine storms and antiviral responses [27]. A different study using nasopharyngeal swabs from infected patients characterized an lncRNA, LASI, that is antisense to the ICAM-1 gene and had an expression profile strongly correlating with SARS-CoV-2 viral loads. LASI’s mechanism of action was shown to be through its interaction with the viral genome and subsequent knock-down of viral replication processes, which also resulted in the presence of inflammation markers [28]. In another study, a pattern of lncRNAs (NRIR, BISPR, and MIR155HG) was observed from nasopharyngeal COVID-19 patient samples that correlated with viral loads [29]. Interestingly, NRIR and BISPR lncRNAs were also upregulated in the blood of COVID-19 patients as reported in the COVIDOME project [30], suggesting possible involvement in the systemic manifestations of the viral infection.

## circRNAs

Circular RNAs (circRNAs) are covalently closed RNA molecules generated by non-canonical back-splicing events from coding and non-coding transcripts. Several studies on the role of circRNAs in viral infections, such as SARS-CoV-2, have been published recently; however reports showing the involvement and function of circRNAs with SARS-CoV-2 infection are few and need to be considered with caution due to the limited number of samples and the intrinsic characteristics of the infection models. In one example, a lung epithelial cell model infected with SARS-CoV-2 showed differential circRNA expression upon infection. Analyzing these circRNA parental genes revealed they were involved with inflammation and immune responses [31]. CircRNAs related to SARS-CoV-2 infection have also been shown to be detectable in the peripheral blood circulation, highlighting their potential as COVID-19 biomarkers [32].

**Fig. 2:**
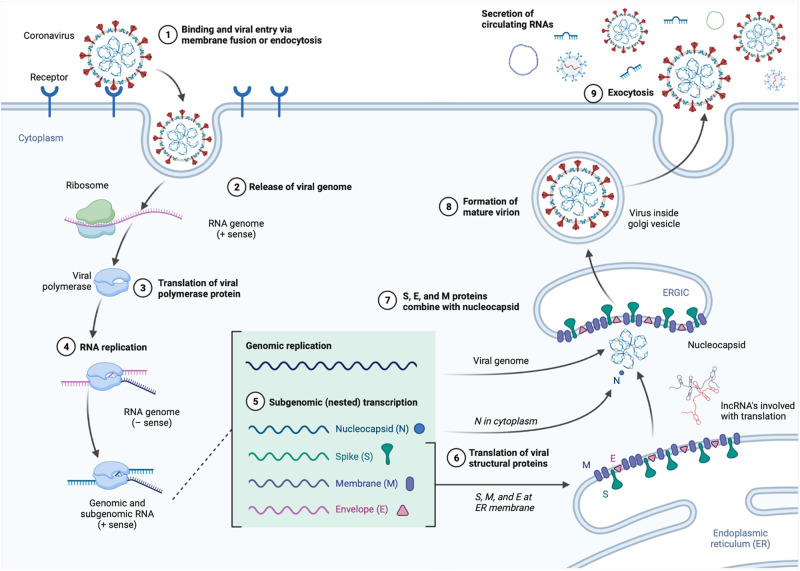
SARS-CoV-2 Intracellular Replication and Extracellular Release of Circulating Factors. An overview of the processes involved with SARS-CoV-2 infection and replication that lead to cellular adaptations (see ref. [3] for more details) and extracellular release of various biological factors into the circulation causing systemic physiological effects.

In this section, we describe humoral factors (and in the previous, metabolism related components) involved in steady-state physiology and responding to SARS-CoV-2 infection in COVID-19 patients, with these intrinsically affecting organ structure and function, and extrinsically affecting systemic physiology based on their production, secretion, and distribution through the circulatory system (Fig. 2). The circulatory system, part of the cardiovascular system, in of itself adapts to SARS-CoV-2 infection, which we will describe in the next section.

## Cardiovascular adaptations and changes

The cardiovascular system includes the heart, arteries, veins, capillaries, and lymphatics, which are the organ systems responsible for the transport of blood, lymph, and their constituents to every organ in the body. Blood and lymph transport multiple components, including water, red blood cells, white blood cells, macromolecules (e.g., lipids, proteins, carbohydrates, macromolecule, etc.), micromolecules (e.g., nutrients, ions, etc.), of which are circulated systemically and transported to and from all organs of the body. Included in the transport of these components by blood and lymph are pathogens, such as the SARS-CoV-2 virus, which benefits from the cardiovascular system not only by binding to a cell-type expressing its cognate receptor (e.g., endothelial cells and ACE2 receptor), but also from the cardiovascular system’s function in transporting the virus and its replicants away from the host infected cell, tissue, and organ. In turn, the COVID-19 cardiovascular-related symptoms vary greatly, ranging from asymptomatic or mild respiratory symptoms to severe life-threatening respiratory and cardiac failure [33].

Early reports have described common symptoms of COVID-19 to include fever (88%), fatigue (38%), chills (11%), headache (14%), nasal congestion (5%), sore throat (14%), dry cough (68%), dyspnea (19%), nausea/emesis (5%), diarrhea (4–14%), and myalgias (15%). A meta-analysis of eight studies showed a higher prevalence of hypertension (17 ± 7%) and diabetes mellitus (8 ± 6%) followed by cardiovascular disease (5 ± 4%) in COVID-19 patients [33, 34]. Moreover, concomitant cardiovascular (CV) conditions may be occurring in 8–25% of COVID-19 infected population and an even greater proportion of those who die from the disease [33]. Indeed, evidence suggests that among the primary causes of death and overall mortality from COVID-19 infection are cardiac issues and respiratory failure [34].

These symptoms result from changes occurring at the molecular level from SARS-CoV-2 infection. A large autopsy study investigating multiple organs (heart, lung, kidney, liver, and lymph nodes) showed that the cellular and functional gene signatures from each organ’s major cell types (e.g., cardiomyocytes in the heart) decrease as a function of the viral load. More specifically in the heart (*n* = 41 from patient autopsy samples), despite no histological change, many heart functional markers (e.g., TNNI3, TNNT2, MYBPC3, PLN, and HAND1) showed decreased levels resulting from COVID-19 infection [35]. Another study showed the presence of SARS-CoV-2 in the myocardial heart tissue from autopsy cases, suggested to be tied to myocardial injury [36]. Indeed, these cellular adaptations extend into the vascular system (e.g., arteries, veins, etc.), with histopathological evidence from autopsy studies showing direct viral infection of endothelial cells and micro- and macrovascular thrombosis occurring in both the arterial and venous circulations. An autopsy study investigating COVID-19 in three patients showed SARS-CoV-2 viral components present in the endothelial cells from different autopsied organs, including the kidney, lung, heart, and liver were each infected with SARS-CoV-2 [37]. Inflammation of the endothelium was also observed, along with recruitment of neutrophils and mononuclear cells to the site of damage. In vitro studies also support SARS-CoV-2 being able to directly infect cardiovascular cells as seen from experiments showing viral infection of engineered human blood vessel organoids [38]. Severe COVID-19 has also been associated with endothelial specific changes and effects such as elevated endothelial activation, hypercoagulability, and increased presence of von Willebrand Factor [39, 40]. Endotheliitis from COVID-19 can occur, leading to a loss of cell–cell junctions, loss of endothelial cells, exposure of the basement membrane, abnormal cytokine release, abnormal reactive oxygen species release, and intussusceptive angiogenesis. Endothelin-1 has been suggested as a possible biomarker for those at risk with COVID-19 [34].

These cardiovascular adaptations from SARS-CoV-2 infection lead to a number of pathophysiological consequences including hypoxia, with prolonged ischemia leading to formation of hypoxanthine as an ATP breakdown product [36]. These effects may be from tissue remodeling caused by viral infection, as well as changes in blood viscosity, clotting, or inflammation, though more research of these areas would benefit our understanding of the effects of SARS-CoV-2 on the cardiovascular system and organ remodeling [33, 34, 36]. For example, a meta-analysis showed there are diverse cardiovascular complications including cardiac injury (e.g., myocardial injury, microinfarcts), heart failure, microembolic infarcts, acute coronary syndrome, arrhythmia, hypertension, and atrial and ventricular complications (e.g., fibrosis) [33, 34]. Autopsy and case reports from COVID-19 patients are also varied in their observations of cardiac tissue changes, highlighting the complexity of the disease and individual differences influencing pathophysiological progression and outcomes. Identifying cardiovascular specific biomarkers would help evaluate, as well as measure progression of disease, with troponin I as one example of a clinical marker evaluating myocardial injury is troponin I. Troponin I has been shown to be elevated in >10% of patients hospitalized with COVID-19, increasing with disease severity [41]. Identifying more markers with cardiovascular specific outcomes (i.e. metabolites, lipids, humoral factors, etc., mentioned in the above sections) would help evaluate, correlate, and triage patients and improve their outcomes, as well as facilitate identification of specific biochemical pathways that may mechanistically explain disease symptoms.

Indeed, with the metabolic and physiological adaptations seen with COVID-19 patients, understanding mitochondrial adaptations in these contexts is relevant, given mitochondrial function in bioenergetics. As discussed in our first review, the mitochondria is heavily dysregulated and suppressed in the host during SARS-CoV-2 infection [3]. One study specifically analyzed transcriptomic data on heart samples collected from deceased COVID-19 patients [6]. This analysis showed heavy suppression at the transcript level of the majority of mitochondrial pathways and genes [6]. The non-coding transcriptome response to SARS-CoV-2 may also play a significant response and regulatory role with the cardiovascular adaptations developing from COVID-19 disease. Whether miRNA’s (e.g., hsa-miR-2392), lncRNA’s (e.g., NRIR and BISPR), or circRNA’s that are involved in suppressing mitochondrial gene expression, changing oxidative phosphorylation, mitochondrial structure (e.g., translation, metabolism, etc.), and causing inflammatory effects, may be involved with local cardiac adaptations or systemic vascular adaptations is unknown, but warrants future investigations to explore the specific roles of the transcriptome with cardiovascular adaptations. Furthermore, given the changes in cardiovascular structure, function, biochemical adaptations, that occur from SARS-CoV-2 infection, further study of cardiovascular mitochondrial adaptations is of relevance, and would help elucidate the underlying mechanisms impacting the cardiovascular system resultant from SARS-CoV-2 infection.

## Immune adaptations and changes

The immune system includes the innate and adaptive immune systems. The innate system involves nonspecific responses to injury, and includes cell populations such as monocytes, macrophages, neutrophils, basophils, eosinophils, etc. The adaptive immune system involves specific response to pathogen infection, and includes cell populations such as T and B cells. The two arms of the immune system communicate, interact, and support each other in responding to injury, aided by humoral, circulating factors such as cytokines and chemokines, that provide information and context to immune cells as to how, where, and what to respond to, as well as the magnitude of the response. In turn, there are various feedback loops that provide guidance and direction to the immune system, both innate and adaptive, with SARS-CoV-2 activating both the innate and adaptive immune systems.

For the innate immune system, activation appears to be guided by neutrophils, which display increased levels of ROS and activated NETosis [42]. Additionally, the loss of ACE2 and DABK in the lungs of patients encourages neutrophil infiltration and inflammation. SARS-CoV-2 has also been suggested to dysregulate type 1 interferon signaling, which contributes to immune evasion early in infection. Consistent with this finding, bulk analysis of whole blood cell transcriptomes of severe COVID-19 patients compared with non-critically ill COVID-19 patients showed upregulation of Interferon alpha as the primary contributor to severe COVID-19. Activated monocytes and macrophages also migrate to the lungs (chemotaxis), further exacerbating lung inflammation and pulmonary fibrosis [43]. Several studies have reported disordered structure of the infected lung, along with extensive immune infiltration [35, 44]. For example, the lung alveoli in COVID-19 patients develop additional hyaluronan, forming a coating that can reduce oxygen exchange, as well as leading to pulmonary edema. Another study of COVID-19 patients showed chronic lung impairment accompanied by persistent respiratory immune alterations, showing specific memory T and B cells enriched at the site of infection [45]. With the significant neutrophil, macrophage, T and B cell infiltration and activation observed, and localized hyperinflammatory states of these immune cells around alveolar epithelial cells, this develops into more lung damage and loss of compartmental identity. When a timeline and pattern of the cytokine storm in tracheal aspirate samples was organized and reported, divergences in the systemic versus airway cytokine production during COVID-19 were observed [46]. Indeed, a study assessing lung function of critical COVID-19 patients showed 55% of them experienced persistent impairment three months after ICU discharge, including declines in patient lung function, exercise capacity, and quality of life [47]. This suggests respiratory failure is another significant factor involved with patient mortality with severe SARS-CoV-2 infection, with an investigation of single-nuclei RNA sequencing of COVID-19 patient lung samples identified substantial alterations in cellular composition, transcriptional cell states, and cell-to-cell interactions, with the data showing COVID-19 patient lungs were highly inflamed, with dense infiltration of aberrantly activated macrophages, though with impaired T-cell responses and suggestions of impaired lung regeneration, based on alveolar type 1 cell phenotypes [48].

With regard to adaptive immune cell responses to SARS-CoV-2, these include lymphopenia (lowered CD4 and CD8 T-cell number) in COVID-19 patients, also seen in autopsy reports [49, 50]. There are case by case variations; for example, an influx of T cells and myeloid cells occurs into the myocardium. In response to this event, COVID-19’s pathogen-associated molecular patterns and damage-associated molecular patterns (PAMPs and DAMPs) are activated, as well as cytokines released by alveolar macrophages [51, 52]. These pathways are thought to contribute to the dysregulation of monocytes and macrophages in COVID-19 [53] and the subsequent dysfunction of T cells. Changes also occur within the heart, as seen with gene expression analysis of cardiac tissue for TNF-ɑ, interferon-γ, chemokine ligand 5, interleukin-6, interleukin-8 (CXCL8), and interleukin-18. Severe COVID-19 is also associated with elevated endothelial activation, hypercoagulability, and the presence of von Willebrand Factor (vWF) [53, 54]. Indeed, COVID-19 induces a complex pattern of cytokine release as part of an overall dysregulated immune response that, in severe cases, injures the parenchyma of vital organs and leads to viremia and sepsis [55, 56].

Severe COVID-19 is characterized by an inflammatory profile that involves initial immune evasion by downregulation of Type I Interferon pathways along with significant upregulation of IL-6, IL-8 and TNF-α in the lungs that coincides with increased NF-κB activity [57, 58]. In one study of COVID-19 patients, a notable increase in multiple serum cytokines (e.g., TNF-α, IFN-γ, IL-2, IL-4, IL-6 and IL-10) was seen [59], while another study showed high plasma levels of IL-1**β**, IL-1RA, IL-7, IL-8, IL-9, IL-10, bFGF, GCSF, GM-CSF, IFN-γ, IP-10, MCP1, MIP-1α, MIP-1**β**, PDGF, TNF-α, and VEGF. A different study showed increased levels of inflammatory markers such as C-reactive protein, D-dimer, ferritin, IL-6 and lactate dehydrogenase (LDH) associated with higher mortality in COVID-19 patients [60]. These results suggest an overproduction of cytokines in response to lung damage, or as a compensatory response to attempt to re-direct, accelerate, or shutdown the immune response. These cytokine fluctuations also coincide with systemic inflammation and myocardial injury; in 137 patients admitted for COVID-19 disease in Hubei province, heart palpitations were noted as one of the presenting symptoms in 7.3% of patients [61]. Moreover, there have been reports of complete heart blockage and atrial fibrillation in patients with COVID-19 infection [62]. In patients diagnosed with severe COVID-19, increased levels of pro-inflammatory cytokines, (e.g., soluble IL-2R, IL-6, IL-8, and TNF-α) have been observed, with these cytokines disrupting endothelial cells antithrombotic and anti-inflammatory functions, leading to coagulation dysregulation, complement and platelet activation, and leukocyte recruitment in the microvasculature [63, 64]. While increased levels of circulating chemokines, pro-inflammatory cytokines, regulatory cytokines, and growth factors were observed in severe COVID patients who died as compared to surviving patients, a more restricted and selective increase of cytokines such as IL-10 was observed in the airway samples with actual decreased levels of pro-inflammatory cytokines such as IFN-γ, IL-17, IL-5, and IL-2 as compared to uninfected controls [46]. These results demonstrate that inflammation is also highly modulated in the airways as an attempt to decrease exacerbated inflammation. Figure 1 illustrates a snapshot of extracellular factors involved with immune adaptations during COVID-19 and the uniqueness of systemic versus airway inflammatory milieu.

## Organ alterations from SARS-CoV-2 infection

**Fig. 3:**
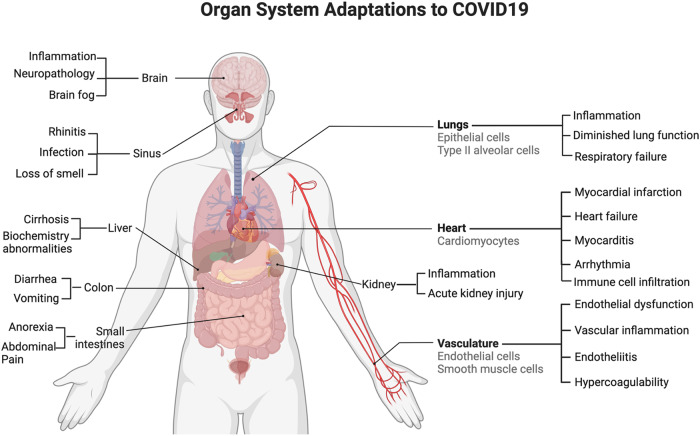
COVID-19 organ adaptations and symptoms. An overview of the systemic organ adaptations resulting from SARS-CoV-2 infection as well as symptoms that develop.

In addition to the cardiovascular system, SARS-CoV-2 infects and has an effect on many organ systems (e.g., cardiovascular, immune, and endocrine systems, as described above), including the lungs, lymph nodes, brain, liver, kidneys, gastrointestinal tract, testis (Fig. 3) [65–67]. COVID-19 is known to cause multi-organ dysfunction, though the burden of infection outside the respiratory tract and time to viral clearance is not well understood. A recent postmortem study of 44 patients quantified and mapped the distribution, replication, and cell-type specificity of SARS-CoV-2 across the body, specifically the brain but other organs as well, from acute infection to more than seven months following symptom onset. It was seen that SARS-CoV-2 infection of the body was widely spread across respiratory and non-respiratory tissues (e.g., the brain) [68]. This spread of infection provides context for the different symptoms patients experience.

Neurological and psychiatric symptoms have been reported for acute, post-acute, and longer-term SARS-CoV-2 infection in human patients across the human lifespan [69]. Although neurological manifestations remain incompletely characterized in terms of etiology, severity, and incidence, they present signs of nonspecific (e.g., headache, dizziness, myalgia, fatigue, somnolence, insomnia, etc.) and specific (e.g., encephalopathy, encephalitis, stroke, status epilepticus, ataxia, anosmia, ageusia, demyelinating syndromes, etc.) deficits of the central nervous system, the peripheral nervous system, or both, with new-onset or relapsed associated prodromal, subthreshold, or fully expressed (i.e., suprathreshold) psychiatric conditions. The spectrum of presumed virogenic psychiatric disorders across age cohorts and other demographic classifications is broad and includes anxiety, depression, confusion or disordered thought (e.g., brain fogor psychosis). Structural and functional changes in brain anatomy, such as brief to protracted alterations in gray and white matter volume, blood perfusion and oxygenation, and metabolic activity [70], often parallel and are consistent with onset and duration of mental health diagnoses in infected populations. Areas caudal to the neocortex tend to be most affected, with olfactory cortex, basal ganglia, hippocampus, amygdala, cerebellum, and brain stem abnormalities occurring in greater frequency and magnitude in COVID-19 patients. Uncertainty regarding the cause of COVID-19-related neuropathology centers on SARS-CoV-2 neuroinvasion, with the olfactory epithelium as a plausible entry point with subsequent retrograde transsynaptic propagation to other brain areas.

Astrocytes and Schwann cells infected by SARS-CoV-2 may also be negatively impacted with their neurotransmission, synapse modulation, reinnervation, metabolite and electrolyte homeostasis, and cell signaling functions. This may cause, contribute, or lead to observed central and peripheral nervous system trauma and dysfunction [70]. There is data that also supports hematogenous mechanisms enabling viral access to nervous tissue through infection of blood-brain-barrier (BBB) endothelial cells or epithelial cells of the choroid plexus blood–CSF barrier. Indeed, a study examining SARS-CoV-2 interaction with brain organoids suggests a compromised blood-brain-barrier (BBB) as manifested by injured endothelial cells and increased BBB permeability [71]. Multisystem hyperinflammatory immunoreactions most appropriately describe for the diverse set of pathologies observed in post mortem autopsy analyses, as well as in live in vivo or in vitro tissue, the result of hemorrhagic, ischemic, and hypoxic injury, mitochondrial dysfunction, neuronal damage and apoptosis, and microglial and astrocytic activation.

With regard to gastrointestinal (GI) effects of SARS-CoV-2, an autopsy study of 29 cases showed the GI to be one of the primary organs infected by the virus, as well as being supported by the literature of postmortem organ analysis [65]. GI involvement and symptoms (e.g., diarrhea, anorexia, nausea/vomiting, abdominal pain, etc.) is associated with long hospital stays and severity of disease, with the rates of GI symptoms of the percent of individuals ranging from 11% to 53%. ACE2 is frequently found in human intestinal epithelial cells, with SARS-CoV-2 being detected in patient GI samples [72]. Indeed, while there is a paucity of data of SARS-CoV-2 effects on GI physiology, GI symptoms are common in patients with COVID-19, though and should be considered when evaluating patient conditions as well as treatment options.

Here we summarize a few examples of organ adaptations resulting from SARS-CoV-2 infection. These highlight the virus’s diverse biological targets and outcomes, though more studies are warranted given our comprehensive lack of understanding of these, and other, organ alterations, as well in particular, the biochemical pathways that are affected by viral infection. In addressing these knowledge gaps, patient care will improve as well as new treatment modalities will be identified. Moreover, these adaptations are related with long-term implications of SARS-CoV-2 infection, of which we describe in the next section.

## Impact of circulating factors on post-acute sequelae of SARS-CoV-2 (PASC, long COVID-19)

In addition to COVID-19 from SARS-CoV-2 infection, patients may experience long-term symptoms as a result of organ or tissue damage due to SARS-CoV-2. This condition is referred to as post-acute sequelae of SARS-CoV-2 (PASC), or long COVID-19 [73, 74]. PASC patients are burdened with multiple symptoms, including more than a third of PASC patients continuing to experience lung fibrotic abnormalities following hospital discharge [75]. Many are also at risk of developing microemboli due to maladaptations with clotting physiology (e.g., massively increased levels of vWF which leads to increased platelet activation, etc.) [76]. Indeed, virus-driven cellular alterations have been suggested to contribute to the pathophysiology of PASC patients [3, 77]. Persistent viral presence and inflammation within the olfactory epithelium has been evident from histological analysis, as well as long-term impairments in taste and smell in the cells of the tongue’s taste buds [78].

PASC is also characterized by chronic fatigue, which has numerous underlying causes. As a comparison, the post-flu or fever fatigue that occurs (e.g., Myalgic Encephalomyelitis/Chronic Fatigue Syndrome (ME/CFS)) may have some similarities to the persistent fatigue in PASC. PASC symptoms may also be influenced by abnormalities of the central nervous system, such as brain hypometabolism [79]. A recent postmortem study also quantified and mapped the SARS-CoV-2 organ tropism, finding long-term signs of infection in the brain, as well as across the body within the tissue (e.g., respiratory, cardiovascular, lymphoid, gastrointestinal, genitourinary, endocrine, skin, and peripheral nervous systems) seven months following infection onset, confirmed through in situ hybridization, immunohistochemistry, and immunofluorescence analyses of the tissue, isolation of SARS-CoV-2 from tissue by cell culture, and detection of the spike sequence variants in non-respiratory sites [71]. While psychological and environmental factors are likely to be involved, long-term biochemical adaptations, including increased oxidative stress and muscle mitochondrial failure, may contribute to the chronic fatigue syndrome found in PASC patients [80]. In addition, due to the activation of the fibrosis pathway from persistent virus-induced inflammation, the heart may subsequently undergo structural remodeling that may also explain the PASC symptoms [81]. There are also long-term immune changes, with PASC patients having elevated expression of type I IFN (IFN-) and type III IFN (IFN-1), highly activated innate immune cells, while lacking naive T and B cells [82], which may also be related with the symptoms PASC patients experience, but also increase their risk of infection, etc.

Indeed, while many individuals recover from SARS-CoV-2, a significant number of patients continue to suffer with long-term disease symptoms (e.g., PASC) long term after recovery from the primary infection [73, 74]. The severity of the primary infection appears to be independent from the possibility and severity of long-term symptoms, though this requires further research. Indeed, it is critical to understand the systemic effects of PASC, its biological mechanism and symptoms, and to develop treatments that promote functional recovery in symptomatic individuals, given the negative health-effects of PASC, as well as the socioeconomic and healthcare burden this condition will impose. Further research on PASC will improve our knowledge and understanding of the condition, as well as enable identification of relevant treatments and interventions to improve patient outcomes.

## Conclusion

SARS-CoV-2 and its resultant acute and chronic diseases, COVID-19 and PASC, have caused significant impacts worldwide. Here we describe and review the latest knowledge about the viral effect on our physiology, as well as the latest research on relevant whole-body metabolism pathways, circulating biological components (e.g., RNA, cytokine, hormones, etc.), and affected organ systems and their physiological adaptations consequent to SARS-CoV-2 infection.

**Fig. 4:**
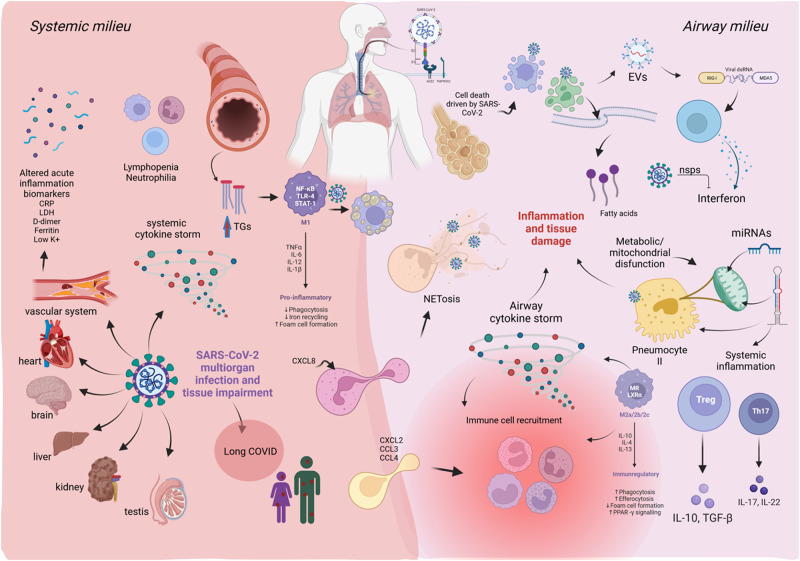
COVID-19 biological adaptations and effects. The multi-faceted and multiplex physiological adaptations resulting from SARS-CoV-2 infection, including the different cells, tissues, and organs that respond to viral infection. The airway milieu refers to the viral factors contained from inhalation and response to these factors in the lungs, while the systemic milieu refers to the viral factors transported through the circulation system and across body, and in turn, the physiological responses to these factors.

COVID-19 is a pleiotropic, complex, and dynamic condition (Fig. 4). The viral insults and cellular adaptations differ depending on the context, from cellular to organ systems, and intricate biochemistry ties and integrates these systems together. Herein, we have attempted to capture the prevalent host responses, as well as highlight gaps of knowledge that could be addressed with future research. We have also touched on relevant biochemical targets with potential for mitigating COVID-19 in the human host, and we will describe current and in-development treatment modalities that affect these in Part 3 of our review.
